# High Heels

**Published:** 1884-07

**Authors:** 


### HIGH HEELS.

SINCE the high heel made its appearance, medical men have more than once borne witness to its bad effects. The late Mr. Hilton condemned it. Others have done the same. Of late years, public opinion has done away with certain of the long-established extravagances of dress, and has given rise to methods more agreeable to the symmetrical development of the body. We hope that in the process of reform the feet, in which too often vanity pays a price which is dangerously expensive, will not escape notice. The evils of the high-heeled boot or shoe are due to the fact that it is an essentially badly fitting article. It is made in defiance of the relation which it ought to bear to the anatomy of the foot, and to the direction in which the pressure of the bodily weight falls upon the latter. Hence the peculiarly cramped walk of ladies of the present day. Any one may observe the consequences of the "advanced position," nearly under the instep, and the increased height of the heel in the substitution of a forward inclination of the body, and a trip suggestive in a measure of the stumbling gait for the upright carriage and the free and graceful swinging movement natural to the leg in walking. These matters, as far as they are merely relative to deportment, do not strictly concern us, but there are attendant circumstances which deserve comment.

The boot or shoe, in order that it may not shift on the foot, which has lost much of its usual purchase of direct downward pressure, must hold it firmly and even tightly, and in particular it is necessarily constructed so as to hold with undue firmness just above the back of the heel. With some persons, perhaps, no inconvenience results; with others who have fine skins chafing is readily produced. This is in itself a trifle, and is presumably altogether too inconsiderate to affect the will of fashion, but it may nevertheless be the slight beginning of graver troubles. Probably there is no practitioner sufficiently long acquainted with town practice who cannot recall a case or cases in which extensive inflammation of the leg, with abcess formation, has followed even a slight abrasion, and the exciting cause, when looked for was discovered in the patient's shoe. There have even been instances, fortunately rare, but still occasional, where abscesses arising from some neglected trifle of this kind have ended fatally.

These are facts that cannot be denied, and should not be overlooked; but even if they could, is there any woman with a mind of her own who will say that the dainty step so much desired by some, bought as it is at the cost of healthy muscular exercise, is not overvalued? We rather hope that honest feeling and the sound judgment which have guided that sex in many better purposes will ultimately overcome the false sentiment which now leads certain of its members to support an unbecoming and injurious custom.—*Lancet.*

